# A Proposed Role for Lymphatic Supermicrosurgery in the Management of Alzheimer's Disease: A Primer for Reconstructive Microsurgeons

**DOI:** 10.1055/a-2513-4313

**Published:** 2025-01-30

**Authors:** Joon Pio Hong, Wei F. Chen, Dung H. Nguyen, Qingping Xie

**Affiliations:** 1 Department of Plastic Surgery, Asan Medical Center, University of Ulsan, Seoul, Korea; 2 Cleveland Clinic, Center for Lymphedema Research and Reconstruction, Cleveland, Ohio; 3 Stanford Women's Cancer Center, Palo Alto, California; 4 Qiushi Hospital Hangzhou, Hangzhou, People's Republic of China

**Keywords:** Alzheimer's disease, glymphatic system, meningeal lymphatic system, intramural periarterial drainage, lymphatic reconstruction, lymphovenous anastomosis, supermicrosurgery

## Abstract

The relatively recent discovery of a novel lymphatic system within the brain meninges has spurred interest in how waste products generated by neurons and glial cells—including proteins associated with Alzheimer's disease (AD) pathology such as amyloid beta (Aβ) and tau—are disposed of. Evidence is building that suggests disease progression in AD and other cognitive impairments could be explained by dysfunction in the brain's lymphatic system or obstruction of drainage. An interesting implication of this hypothesis is that, by relieving the obstruction of flow, lymphatic reconstruction along the drainage pathway could serve as a potential novel treatment. Should this concept prove true, it could represent a surgical solution to a problem for which only medical solutions have thus far been considered. This study is meant to serve as a primer for reconstructive microsurgeons, introducing the topic and current hypotheses about the potential role of lymphatic drainage in AD. A preview of current research evaluating the feasibility of lymphatic reconstruction as a surgical approach to improving Aβ clearance is provided, with the aim of inspiring others to design robust preclinical and clinical investigations into this intriguing hypothesis.

## Introduction

The progression of Alzheimer's disease (AD) is a complex pathological process characterized by heterogeneous clinical presentation, level of cognitive impairment, and neuropathologic characteristics. 1 2 Memory loss and confusion are perhaps the most familiar manifestations of the disease, but people with AD can also exhibit a spectrum of deficits in language, visuospatial perception, behavior, and praxis that correlate with the specific region of the brain affected by atrophy. 1 2 3

While AD is well known for its hallmark neuropathology—the abnormal accumulation, aggregation, and deposition of the neurotoxic AD protein biomarkers amyloid beta (Aβ) and tau 4 —the primary events that precipitate disease onset and lead to its progression have not been established definitively. AD is a complex puzzle; genetic factors as well as disruptions in cholesterol metabolism, inﬂammation, and even intracellular functions have increasingly been implicated in AD pathogenesis and progression, but the causal relationships between these many processes, and their potential utility as therapeutic targets, are not completely understood. 1 2 3

An emerging concept that has particular relevance to the practice of lymphatic surgery is the potential connections between AD neuropathology, the physiologic and anatomical mechanisms of central nervous system (CNS) waste clearance, and lymphatic drainage. 5 6 7 8 9 10 11 12 13 14 15 Several lines of evidence have together suggested that AD could, at least in part, be a disorder of lymphatic obstruction that hinders clearance of Aβ and other neurotoxic products from the brain, thus making surgical improvement of lymphatic drainage an intriguing and novel therapeutic target. 5 6

Previously, AD and related pathologies have been approached as problems to be solved medically; should research confirm a role for lymphatic obstruction in the pathogenesis of AD, then it suggests the potential for a surgical solution requiring microsurgical techniques and expertise. Therefore, this study is written to introduce reconstructive microsurgeons to the key concepts and to inspire scientific curiosity.

We provide a brief overview of brain anatomy and new insights into the presence of a brain-specific lymphatic system and study the evidence supporting the idea that the mechanisms for clearing toxins from the CNS connect to, and drain through, this lymphatic system into the deep cervical lymph nodes (dcLN). We then summarize current efforts to demonstrate that lymphovenous reconstruction can improve waste clearance from the brain as well as clinical outcomes in AD. Finally, we present our own hypothesis that supermicrosurgical, side-to-end anastomosis is the surgical technique of choice to improve drainage and the clearance of potentially toxic waste products, including Aβ and tau, from the brain.

## Overview of Cerebral Clearance and Drainage: An Evolving Understanding

The brain is the most metabolically active organ of the human body, 16 producing numerous waste products that collect within the interstitial fluid (ISF) that bathes the neurons and glial cells of the brain parenchyma. 17 These include byproducts of metabolism, cellular debris, degraded or misfolded proteins, and other macromolecules. Throughout the rest of the body, interstitial debris and toxins would be directed through the lymphatic system into the venous circulation for processing and clearance, but it has long been understood that a conventional, histologically identifiable lymphatic vasculature does not exist within the brain parenchyma. 18 19 20

Nevertheless, the mammalian brain does have mechanisms for waste clearance and drainage. Waste products in the ISF within the brain parenchyma are exchanged with cerebrospinal fluid (CSF), which fills the compartmentally distinct cerebral ventricles and subarachnoid space of the brain meninges (membranes that enclose the CNS). 20 21 22 Until recently, the prevailing hypothesis was that the solute exchange between ISF and CSF occurred through the convective bulk flow of the ISF, 23 24 25 and that the CSF carrying waste products eventually passed through the cervical lymph nodes (cLN) to be reabsorbed into the bloodstream. 20 Some of the proposed routes for reabsorption included the arachnoid villi within the dural sinuses, along cranial nerve sheaths, through the olfactory lymphatics, or the dcLNs. 24 26 27 28

Discoveries made within the last decade have changed our understanding of the waste-clearance process significantly. Novel, brain-specific anatomical and functional systems have been discovered that suggest that the cerebral drainage pathway includes a paravascular glial-lymphatic (glymphatic) system, paired with a novel lymphatic network that permeates the meninges and ultimately drains CSF and waste products to the dcLN. These findings have important implications in developing treatment strategies for age-related and neurodegenerative diseases.

### The Glial-Lymphatic (Glymphatic) and IPAD Systems: Competing Hypotheses with a Common Endpoint

The concept of a paravascular space within the brain parenchyma has long been theorized, 20 but in 2012, Iliff and colleagues used modern tracer and imaging techniques in mice to fully characterize a paravascular pathway that begins with an influx of subarachnoid CSF along para-arterial spaces infiltrating the brain parenchyma. 19 After solute exchange from ISF to CSF takes place within the capillaries of the neurovascular unit, 29 CSF efflux occurs along the paravenous space surrounding the large deep veins. 18 This pathway was termed the “glymphatic” pathway because, while it has functional similarity to the peripheral lymphatic system, it is anatomically distinct and mediated principally by glial cells.

Competing models of parenchymal waste clearance exist, with significant controversy over the specific anatomical space and direction in which waste fluid flows. The most prominent of these alternative hypotheses is the intramural periarterial drainage (IPAD) model, in which waste fluid travels along a periarterial space that is anatomically distinct from the paravascular compartment, with the direction of efflux opposite of that of the paravascular model. 18 30 31 32

Despite these major and unresolved differences in the two leading models for brain-waste clearance, there is evidence that interstitial metabolic waste products and solutes, including Aβ, may be cleared from the brain by one or both of these pathways, 30 32 33 34 35 36 and that the postparenchymal drainage pathway exits the brain through a novel lymphatic system within the meninges. 37 38

### Meningeal Lymphatic System Connects the Brain's Clearance Pathways to the Peripheral Lymphatic System

The meninges are three membranes, separated by fluid-filled spaces that serve to cushion and protect the brain and spinal cord and maintain fluid homeostasis. 22 The innermost pia mater and the middle arachnoid mater layers enclose the subarachnoid space, which is filled with CSF produced within the cerebral ventricles. Covering these two membranes is the outermost dura mater, which is in direct contact with the skull, and surrounds and supports the dural venous sinuses. The subdural space is a compartment distinct from the subarachnoid space and is filled with serous fluid, not CSF.

In 2015, research groups at the University of Virginia and the University of Helsinki independently published reports identifying and characterizing a novel lymphatic network within the meninges in separate mouse models. 37 38 Aspelund and colleagues showed that meningeal (dural) lymphatic vessels absorb CSF and waste products from the subarachnoid space, and then drain to the dcLN via foramina at the base of the skull. 38 Louveau and colleagues also demonstrated that the meningeal lymphatic vessels (mLVs) communicate directly with the dcLNs and that they, rather than the superficial cLN or the lymphatics of the nasal mucosa, were the primary drainage route into the dcLNs for waste products derived from subarachnoid CSF. 37 In a later study, Ahn and colleagues showed that the basal mLVs exhibited specific morphologic features associated with fluid uptake and drainage, including blunt-ended lymphatic capillaries with button-like junctional patterns, and lymphatic valves that resemble precollectors. 10 They went on to demonstrate that the basal mLVs are the main pathway for clearance of waste-laden CSF into the peripheral lymphatic system.

While these groups were not the first to suggest the presence of a brain-specific lymphatic system, their discoveries confirmed prior hypotheses that had not been accepted previously by the scientific community. 20 Critically, the revelation of these hidden, atypical lymphatic vessels answered the lingering question of how the immunologically privileged compartments holding the CSF and ISF connect to the peripheral lymphatic system.

### CSF and Cerebral Waste Drain through the dcLN

**Fig. 1:**
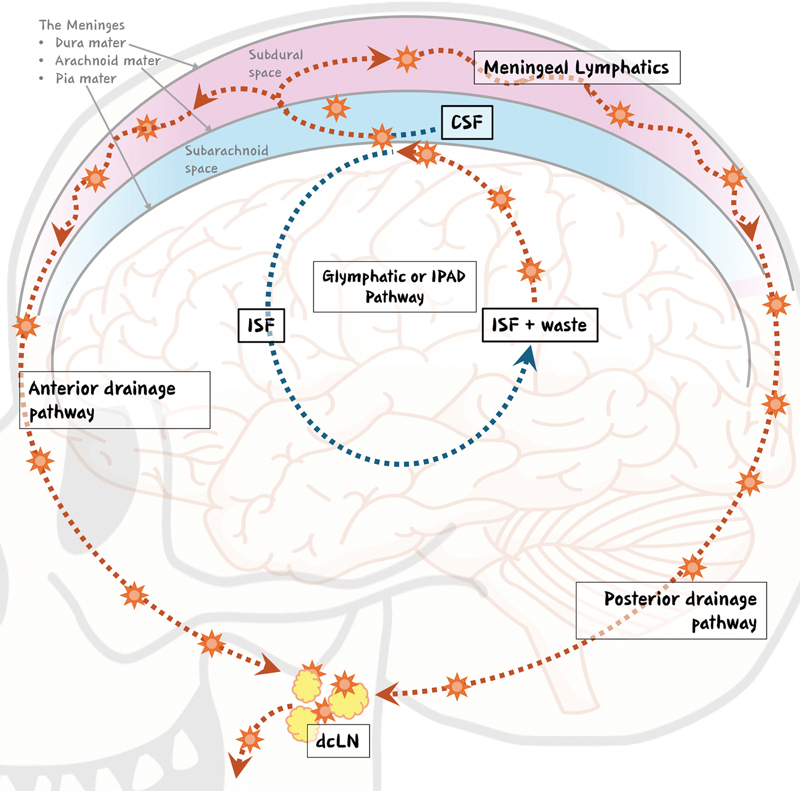
A simplified schematic of the waste clearance and drainage pathway. Waste products such as metabolic products, cellular debris, and degraded or misfolded proteins are collected in the interstitial fluid (ISF) within the brain parenchyma. Through an as-yet debated pathway, the waste is exchanged between the ISF and cerebrospinal fluid (CSF) in the subarachnoid space. The CSF and waste then travel into the meningeal lymphatics, which drain to the deep cervical lymph nodes and then out to the peripheral circulation. (The images of the skull and brain were designed by Freepik and are available for free.)

In summary, whether the exact anatomical pathway by which waste-laden CSF enters the lymphatic system is from the glymphatic or IPAD pathway, for the purposes of the plastic and reconstructive surgeon, the end is the same ( Fig. 1 ). Extracellular Aβ and other macromolecules and inflammatory mediators are carried away from the cerebral ISF by the subarachnoid CSF, exit the brain through the basal mLVs, drain directly into the dcLNs, and then enter the peripheral venous circulation through the jugular lymphatic trunk. 10 37 38 39

This summary is an oversimplification of a vastly complicated system that depends on the proper functional interaction of numerous cell types, membrane proteins, anatomical structures, and fluid dynamics, many of which are not completely established. For the reader who is interested in a more detailed and nuanced background on these topics, we suggest several recent studies. 17 20 33 35 36 37 38 40 41 42 43 44 45 46

Ultimately, numerous hypotheses based on diverse lines of investigation converge on the concept that the accumulation of the Aβ and tau aggregates that characterize AD, aging, cognitive impairment, and other neurodegenerative conditions result from impairment in the clearance of these macromolecules from the brain and that dysfunctions in clearance may, in part, be attributable to lymphatic dysfunction.

## Evidence Connecting Lymphatic Obstruction and Pathogenesis of Alzheimer's Disease

### Evidence from Preclinical Models

A great deal of the evidence for the brain-waste clearance pathways is derived from studies in normal mice and transgenic or knockout mouse models of AD and other neuropathologies with specific features of Aβ or tau pathology. For example, in 2018, Da Mesquita and colleagues used multiple techniques (photodynamic drugs, surgical ligation, and a transgenic mouse deficient in lymphatic vessel function) to ablate mLV in mice. 13 The results of this perturbation were impaired perfusion of the brain by CSF, as well as an increase of Aβ deposition and inflammatory response within the meninges and hippocampus of a mouse model of AD and in aged mice. The authors concluded that meningeal lymphatic dysfunction may exacerbate AD and age-associated cognitive decline and that improvement in the function of mLV is a potential therapeutic target for these pathologies.

Subsequently, Patel and colleagues used imaging and blood plasma quantification to trace the pathway and clearance rate of dye-labeled tau from the brain parenchyma of wild-type and transgenic mice engineered to lack a functional cerebral lymphatic system. They showed that the brain parenchyma of mice lacking a CNS lymphatic system retained greater amounts of tau, and exhibited significantly delayed clearance of a reference tracer, compared to wild-type controls. 11

Consistent with these studies, Wang and colleagues investigated the effect of blocking the dcLNs, in wild-type mice and a mouse model of AD, using surgical ligation. 12 They found that dcLN ligation in AD mice exhibited increased accumulation of Aβ within the brain, neuroinflammation, synaptic protein loss, impaired polarization of the key glial-cell water channel AQP4, and cognitive and behavioral deficits. The authors concluded that impairment of lymphatic clearance from the brain is an important factor in AD progression and a potential therapeutic target.

Also of interest is a 2019 report from Ahn and colleagues, 10 in which they performed anatomical, morphologic, and functional characterization of basal mLVs in mice. In addition to demonstrating that basal mLVs represent the primary drainage pathway for CSF containing cerebral waste products, they also found that, as mice age, the basal mLVs acquire lymphedematous characteristics with associated impairment in CSF drainage. Together with prior findings that CSF production and turnover have also been shown to slow with aging, 14 the authors speculated that the result is dysfunction and delayed clearance of cerebral waste products leading to increased risk for AD and other age-related disorders of cognitive impairment. 10

Finally, in a 2024 report, Wang and colleagues used noninvasive, near-infrared light to modulate mLV drainage in mouse models of aging an AD. Remarkably, treated mice showed a significant reduction in deposition of Aβ, neuroinﬂammation, and neuronal damage with associated improvement in cognition. The results of this study support prior work suggesting that the exit route for the brain clearance system should be a target of interest for treatment of AD. 7

### Evidence in Humans

Although the majority of studies regarding the existence, structure, function, and connectivity of the proposed para- and perivascular pathways and mLVs have been performed in mice, the presence of these systems in the human brain has been confirmed through imaging and other functional studies. 8 9 15 47 48 49 In 2018, Eide and colleagues demonstrated CSF tracer drainage to dcLNs in humans. 48 This group presented the mechanism of CSF transport to the dural lymphatics, suggesting that the parasagittal dural space serves as a bridging link between the human brain and the dural lymphatic vessels. 50 They also showed that CSF drainage via the cribriform plate is negligible in humans, which is different from the CSF efflux to the nasal mucosa in animals. 51 The identification of dcLNs responsible for CNS waste clearance was made possible through the capture of CSF-draining dcLNs in contrast-enhanced magnetic resonance imaging. 48 This localization of the dcLNs that drain CSF has since been independently verified. 52

Furthermore, in 2022 Nauen and Tronosco confirmed that Aβ is present in human lymph nodes, suggesting that glymphatic clearance of Aβ through the mLV pathway described in rodent models is conserved in the human brain. 9

Recently, Chao and colleagues investigated the incidence of new-onset dementia in patients, aged 60 years or older, who had undergone cLN dissection for treatment of head and neck cancer. 53 Out of a total of 251 patients, 9 of 234 male patients developed dementia, on average within 4.2 ± 2.9 years after surgery. This corresponded with a dementia incidence rate of 0.7 per 100 patient-years and a cumulative incidence of 10.34% over 8.6 years—higher than published incidence rates of dementia in the general population. They also found a significant association between dementia onset and bilateral cLN dissection compared to patients who underwent a unilateral procedure. As a retrospective analysis, the study has a number of limitations including those related to cause and effect; however, the results are another piece of evidence supporting a role for cLN in preserving healthy brain function.

New data from a forthcoming research report from Hong and colleagues has used ultrasound to compare cLN characteristics between 25 healthy patients (258 lymph nodes) and 25 patients (207 lymph nodes) with a positive diagnosis of dementia (manuscript in preparation). A preliminary analysis has found that in zones I–IV, there is no statistically significant difference in the number and shape of cLN between groups. However, the numbers and symmetry of cLNs are significantly different between groups in zone V and, in fact, are asymmetrical between the left and the right sides in dementia patients. The findings provide further indirect evidence that lymphatic obstruction is associated with dementia and suggest that cLNs in zone V that collect drainage from the anterior and posterior pathways should be studied as potential sites for lymphovenous anastomosis (LVA).

## Lymphatic Reconstruction as a Potential Therapeutic Target for Alzheimer's Disease

Given the body of evidence supporting the hypothesis that lymphatic obstruction is a likely contributor to the pathogenesis and/or progression of AD, we and others have proposed that lymphovenous bypass within the dcLNs could potentially improve drainage, clearance, and clinical outcomes. 5 6 Indeed, investigation of the potential efficacy of this kind of bypass for the treatment of cognitive dysfunction is already underway, in both animal models and in human subjects. This year, Xie and colleagues at Qiushi Hospital in the People's Republic of China reported that their group has completed extracranial lymphatic reconstruction in at least 50 patients with AD, with a mean of nine months follow-up. 5 To date they have presented video from one case, in an 84-year-old man with AD, to show his improvement from a bedridden preoperative baseline to marked functional improvement at three days, six months, and eight months postprocedure. We await a full analysis of their findings and details of their procedural approach with great interest.

Chen and colleagues at Cleveland Clinic recently provided a basis for how lymphatic reconstruction may help with AD and how extracranial lymphatic reconstruction may improve intracranial lymphatic dysfunction. Using indocyanine-green imaging in 152 patients with primary lymphedema, they demonstrated that primary lymphedema, despite often presenting with localized symptoms, is a systemic condition affecting the entire body. 54

In a subsequent study of 124 primary lymphedema patients, 32% exhibited cognitive deficits—a rate nearly three times higher than the general population. Notably, patients who underwent lymphatic reconstruction, such as LVA, experienced systemic improvements, including better cognitive function (manuscript in preparation). These patients reported enhanced memory and mental clarity and scored significantly higher on validated cognitive tests compared to those without surgery.

Based on these findings, the team hypothesized that brain lymphatic dysfunction contributes to the elevated incidence of cognitive impairment in primary lymphedema and that lymphatic reconstruction indirectly improves brain lymphatic function. Extending this concept, they proposed that AD, characterized by cognitive decline linked to lymphatic dysfunction, could be treated through extracranial lymphatic reconstruction, which has consistently demonstrated systemic therapeutic effects.

Building on this foundation, Li and colleagues at Shanghai Jiao Tong University School of Medicine recently announced the initiation of an investigator-initiated trial of a novel, extracranial procedure they call “cervical shunting to unclog cerebral lymphatic systems (CSULS).” 6 This approach involves LVA to connect the bilateral deep cervical lymphatic vessels to the low-pressure venous system. While it has not been demonstrated that the brain lymphatic system has a pressure gradient, the venous pressure in the neck is naturally low (between 0 and 6 mmHg) 55 and the effect of gravity is likely to exert pressure on the draining lymphatic system. Therefore, the authors' assumption is that their CSULS procedure will bring about lymphatic trunk decompression and improved drainage, 6 in a manner analogous to that of LVA treatment for peripheral lymphedema.

This group has recently published observations from the first case from this trial in a 76-year-old female patient who met guideline diagnostic criteria for AD. 6 Within 5 weeks after the procedure, favorable changes in Mini-Mental Status Examination score, Clinical Dementia Rating-sum of boxes test, Geriatric Depression Scale score, and objective positron-emission tomography (PET) scan measures were observed, as well as subjective measures from the patient's family. This group has reported that they have performed procedures on six patients and plan to publish their findings once all patients have completed prespecified follow-up milestones.

Nguyen and colleagues at Stanford University are currently investigating the feasibility of a treatment to induce directional lymphatic regeneration and establish new lymphatic pathways using a novel, biodegradable, implantable nanofibrillar scaffold. 56 57 This material has not yet been evaluated for addressing neurological disorders, but has been successfully and safely applied in upper- and lower-limb secondary lymphedema as well as head and neck lymphedema. 58 59 60 61 The potential benefits of using bioengineered scaffolds to promote lymphatic regeneration in the treatment of AD is an intriguing area for further study.

Meanwhile, a collaboration between groups at the Cleveland Clinic and the University of Wisconsin is conducting investigations in an established murine model of AD to evaluate AD biomarkers before and after extra-anatomic, supermicrosurgical lymphatic reconstruction. 5 This quantitative evaluation within a highly controlled preclinical setting will provide much-needed basic evidence with which to interpret findings in human studies.

## Proposed Role for Supermicrosurgery in Brain Lymphatic Reconstruction

We applaud the pioneering work of Xie and colleagues in conducting and extending these studies, which hold promise to provide valuable proof of concept to understand the feasibility of a lymphatic reconstructive approach to AD treatment. As with any surgical innovation, it is the job of those who follow to refine the technique to optimize it for safety and enhance its outcomes. Thus, given our extensive experience with lymphatic microsurgery and the growing field of supermicrosurgery, 56 59 60 61 62 63 64 65 as a group we urge our colleagues to exercise caution with full consideration to the gaps in knowledge that could negatively affect patient safety and the acceptance of novel findings.

Considering the principle of “first, do no harm,” we assert that further pursuit of this hypothetical approach must be conducted through rigorous, systematic science, with preclinical studies and clinical trials designed after a thorough study of the potential merits and drawbacks of lymphovenous reconstruction techniques in the setting of the CNS. Above all, any clinical investigation should prioritize patient safety over a drive to evaluate clinical effectiveness.

A number of critical questions must be considered before initiating new clinical efforts; these include the relative safety and effectiveness of the types of lymphatic dissection, and the number and types of anastomoses that will maximize drainage while preserving flow and distal function in the treated vessels. For example, it is not known whether it is sufficient to dissect the entire posterior bundle *en masse* and connect it to the surrounding superficial veins. The potential complications and downstream consequences of this relatively rudimentary approach are unknown and should be compared to a more refined, supermicrosurgical technique that creates a 1:1 vessel coaptation, intima to intima. The risk of scarring and thrombosis are concerns in this context, and unlike in lymphatic reconstruction of the extremities, the head and neck may lack collateral drainage pathways that could compensate for unsuccessful reconstruction. Indeed, the recent publication from Chao and colleagues demonstrating higher levels of dementia in patients with head and neck cancer who underwent dissection of the cLNs reinforces the wisdom of protecting lymphatic function. 53

There are also numerous mechanical and physiological mechanisms that are not yet understood, including whether the brain lymphatic system has a pressure gradient as has been observed in peripheral lymphatics, or instead relies on gravity for drainage. The presence and function of valves within this system remain unexplored.

Another emerging area to be studied is a comparison of the safety and efficacy of end-to-end versus side-to-end lymphaticovenous anastomoses. 62 End-to-end approaches may be of concern for obstruction, scarring, and loss of distal function, while the concept behind side-to-end anastomosis is that this connection may produce a more favorable pressure gradient, enable both anterograde and retrograde flow, and preserve the downstream distal function of the involved lymphatic vessels. In a recent, retrospective comparison of outcomes, after 123 patients with peripheral lymphedema were treated with either end-to-end (n = 63) or side-to-end (n = 60) lymphaticovenous anastomoses, the side-to-end group experienced a significantly better volume reduction in all time intervals ( *p* < 0.03) and longitudinal outcome ( *p* = 0.004). 62 While early-phase patients showed no difference between the two groups, advanced lymphedema patients in the side-to-end group experienced a significantly better volume reduction ratio at all time intervals ( *p* < 0.025) and on the overall longitudinal outcome ( *p* = 0.004) compared to end-to-end patients.

Therefore, it is our opinion that true supermicrosurgical techniques, performed with precision and efficiency, should be the gold standard for these reconstructions in AD patients. These entail side(lymphatic)-to-end(venous) anastomosis of individual vessels or lymph-node-to-vein anastomosis. 66 These approaches have been proven in the treatment of peripheral lymphedema and we speculate that, in the even more delicate setting of lymphatic surgery aimed at relieving obstruction of drainage from the brain, supermicrosurgery may help to avoid unanticipated complications of a macro-scale dissection and anastomosis. 62 63 64 Again, the aim of using these advanced supermicrosurgical approaches is to preserve remnant lymphatic function and normal, physiologic flow to minimize the possibility of unintended long-term consequences and potential obstruction over time.

## Conclusion and Future Directions

At present, the hypothesis that extracranial lymphovenous reconstruction of any kind could be used to treat AD and related disorders of cognitive impairment remains a hypothesis. While an intriguing base of evidence supports the concept, continued investigation in animal models and rigorously designed clinical studies with appropriate controls and quantitative outcome measures will be required to validate—or refute—the efficacy and safety of this approach. Objective quantitative evidence is needed, including biomarker measurements, imaging studies, and validated neuropsychological evaluations. Extensive work must be undertaken to refine surgical techniques to identify the best possible method for maximal outcomes and minimal complications. Should the research prove fruitful, it could represent a new way of thinking about solutions to the problem of dementia. We encourage our surgical colleagues to monitor and participate in this exciting new direction of study.
